# Editorial: Personalized precision medicine in autism spectrum related disorders, volume II

**DOI:** 10.3389/fneur.2024.1392642

**Published:** 2024-03-18

**Authors:** Lidia V. Gabis, Andreea Nissenkorn, Josephine Barbaro

**Affiliations:** ^1^Pediatrics, School of Medicine, Tel Aviv University, Tel Aviv, Israel; ^2^Keshet Autism Center Maccabi Wolfson, Holon, Israel; ^3^Department of Pediatrics, Wolfson Medical Center, Holon, Israel; ^4^School of Psychology and Public Health, Olga Tennison Autism Research Centre, La Trobe University, Melbourne, VIC, Australia

**Keywords:** autism, infants, precision medicine, fragile X, genetics

Individuals with a diagnosis of Autism Spectrum Disorder (ASD, “autism”) share differences in social attention and communication, and the presence of repetitive and sensory behaviors and/or focused and specialized interests, as defined by the Diagnostic and Statistical Manual of Mental Disorders-5-TR (DSM-5-TR) (1). However, individual differences in specific developmental trajectories linked to autism include different etiologies (genetic and environmental, such as premature birth), different age of identification, diagnosis and cooccurring conditions.

As a result of these individual differences, it is pertinent that a personalized approach is taken to providing supports and services for individual diagnosed with ASD that not only relate to the core traits of autism, but also the management of its many co-occurring conditions. Thus, this Research Topic explores a more personalized approach to the identification, diagnosis, and support of children with autism, commencing in infancy.

In this edition, early identification of autism is discussed in relation to a large, state-wide rollout of training on autism to primary health practitioners, with the aim of providing timely supports and services to children and their families. The study, “Development, delivery, and evaluation of a training program for the early identification of autism: Monitoring of Social Attention, Interaction, and Communication” (MoSAIC; Gilbert et al.) described the development and implementation of the MoSAIC training program for Maternal and Child Health Nurses (MCHNs) in Victoria, Australia, who are in a unique position to monitor infants and toddlers for signs of autism through routine well-baby consultations. The program included online pre-workshop modules, face-to-face workshops, and online post-workshop modules. A training satisfaction survey showed that over 90% of MCHNs found the training clear, of high quality, well-presented, and would recommend it to colleagues. In the 6 months following the training, MCHNs conducted over 82,000 assessments using the Social Attention and Communication-Revised (SACS-R) tool (2) indicating successful integration of early autism checks into their routine practice. This study demonstrated the feasibility, acceptability, and effectiveness of training a large healthcare workforce for the universal developmental surveillance of autism.

A follow-on study entitled “Investigating autism knowledge, self-efficacy, and confidence following maternal and child health nurse training for the early identification of autism” (Gore et al.) aimed to assess MCHNs' competencies in terms of autism knowledge, self-efficacy in identifying autism in infants, and their confidence in discussing autism with parents. Results showed that previous autism training and knowledge of community resources significantly contributed to increased self-efficacy in identifying infants at “high likelihood” of autism, while knowledge of community resources was the best predictor of confidence in discussing autism with parents. The study highlighted the importance of targeted autism training for primary health practitioners to enhance early autism identification, and initiate conversations with parents early and in an affirming manner, with the ultimate aim of providing early supports and accommodations for children with autism and their families.

Tailoring supports for autism with and without co-occurring conditions, is critical. To assist with this, we can investigate the path from etiology to supports within related neurodevelopmental conditions such as Fragile X syndrome and Down Syndrome. In “*A longitudinal investigation of pragmatic language across contexts in autism and related neurodevelopmental conditions*,” the researchers focused on pragmatic language skills in individuals with autism and those with related neurodevelopmental conditions (Martin et al.). The study included boys with “idiopathic autism spectrum disorder (ASD),” fragile X syndrome with and without ASD (FXS-ASD, FXS-O), Down syndrome (DS), and typically developing (TD) boys. Results indicated that boys with idiopathic ASD and FXS-ASD showed the most significant difficulties in pragmatic language across different contexts, with the most pronounced difficulties observed in unstructured conversations. The study highlighted the importance of understanding social language difficulties across various neurodevelopmental conditions and tailoring assessments and supports accordingly.

In the study “*An escalating continuum of learning and attention difficulties from premutation to full mutation in female carriers of FMR1 expansion*” (Gabis et al.), the cognitive effects of Fragile X premutation in females was examined. Findings revealed a correlation between the number of CGG repeats and learning and attention difficulties, impacting daily functioning skills such as driving and handling schedules. Despite these challenges, most carriers of premutation and full mutation functioned well in many areas. This study highlighted the need for specific interventions to address learning difficulties and improve daily functioning linked to premutation of FMR1 gene.

These manuscripts collectively underscore the significance of early autism identification and examining genetic linked trajectories and sex differences in children with autism. It highlights the importance of training of healthcare practitioners on early autism presentation, and tailoring supports for people with autism with and without co-occurring conditions such as Fragile X and Down Syndrome, particularly in understanding and supporting communication and learning differences.

## Author contributions

LG: Conceptualization, Methodology, Supervision, Validation, Writing—original draft, Writing—review & editing. AN: Supervision, Writing—review & editing. JB: Conceptualization, Validation, Writing—review & editing.
